# The impact of ageing on natural killer cell function and potential consequences for health in older adults

**DOI:** 10.1016/j.arr.2013.04.003

**Published:** 2013-09

**Authors:** Jon Hazeldine, Janet M. Lord

**Affiliations:** MRC-ARUK Centre for Musculoskeletal Ageing Research, School of Immunity and Infection, University of Birmingham, Birmingham B15 2TT, UK

**Keywords:** ADCC, antibody dependent cell cytotoxicity, Apaf-1, apoptosis-activating factor 1, BID, BH3-interacting domain, CAD, caspase-activated DNase, CMV, cytomegalovirus, DC, dendritic cell, DLN, draining lymph node, FasL, Fas ligand, FADD, Fas-associated protein with death domain, iCAD, inhibitor of caspase-activated DNase, IFN-γ, interferon gamma, IL-8, interleukin 8, KIR, killer cell immunoglobulin like receptor, MHC, major histocompatibility complex, MIC, MHC class I-chain-related protein, MIP-1α, macrophage inflammatory protein-1-alpha, NCR, natural cytotoxicity receptor, NK cell, natural killer cell, NKCC, natural killer cell cytotoxicity, PARP, poly ADPribose polymerase, PBLs, peripheral blood lymphocytes, PMA, phorbol 12-myristate 13-acetate, TB, *Mycobacterium tuberculosis*, tBID, truncated BH3-interacting domain, TNF-α, tumour necrosis factor alpha, Th-1, T helper 1 cell, TRAIL, tumor necrosis factor related apoptotic-inducing ligand, Natural killer (NK) cells, Ageing, Immunesenescence

## Abstract

Forming the first line of defence against virally infected and malignant cells, natural killer (NK) cells are critical effector cells of the innate immune system. With age, significant impairments have been reported in the two main mechanisms by which NK cells confer host protection: direct cytotoxicity and the secretion of immunoregulatory cytokines and chemokines. In elderly subjects, decreased NK cell activity has been shown to be associated with an increased incidence and severity of viral infection, highlighting the clinical implications that age-associated changes in NK cell biology have on the health of older adults. However, is an increased susceptibility to viral infection the only consequence of these age-related changes in NK cell function? Recently, evidence has emerged that has shown that in addition to eliminating transformed cells, NK cells are involved in many other biological processes such as immune regulation, anti-microbial immune responses and the recognition and elimination of senescent cells, novel functions that involve NK-mediated cytotoxicity and/or cytokine production. Thus, the decrease in NK cell function that accompanies physiological ageing is likely to have wider implications for the health of older adults than originally thought. Here, we give a detailed description of the changes in NK cell biology that accompany human ageing and propose that certain features of the ageing process such as: (i) the increased reactivation rates of latent *Mycobacterium tuberculosis*, (ii) the slower resolution of inflammatory responses and (iii) the increased incidence of bacterial and fungal infection are attributable in part to an age-associated decline in NK cell function.

## Highlights

• Roles are emerging for natural killer (NK) cells beyond removing transformed cells.
• These include immune regulation and the elimination of senescent cells.
• Human ageing is associated with a decline in NK cell function.
• We propose some aspects of human ageing are due in part to reduced NK cell function.
• These include reduced vaccination efficacy and delayed resolution of inflammation.

## 1 Introduction

Comprising 10–15% of the circulating lymphocyte pool, natural killer (NK) cells are large granular lymphocytes of the innate immune system renowned for their ability to recognise and eliminate virally infected, stressed and malignant cells. In humans, NK cells, whose defensive strategies include direct cytotoxicity and the secretion of immunoregulatory cytokines and chemokines, are defined by a CD3^−^ CD56^+^ surface phenotype. However, they are not a homogenous population, as based on the differential surface expression of CD56, NK cells are categorised into two major subsets: CD56^DIM^ and CD56^BRIGHT^, which differ both in their receptor profile and function (Cooper et al., 2001a).

Physiological ageing is associated with changes in the composition, phenotype and function of the circulating NK cell pool, a phenomenon referred to as NK cell immunesenescence (Solana et al., 1999, Solana and Mariani, 2000, Tarazona et al., 2012). As NK cells represent the first line of defence against virally infected cells, immunogerontological studies often introduce or conclude their work by proposing that NK cell immunesenescence contributes to the higher incidence of viral infection that is reported by older adults (Mariani et al., 1996, Rukavina et al., 1998, Mariani et al., 2002a, Hayhoe et al., 2010). However, over the past decade, data has emerged demonstrating that NK cell function extends beyond merely the recognition and elimination of transformed cells, with studies being published implicating a role for NK cells in: (i) anti-microbial defence (Small et al., 2008, Schmidt et al., 2011), (ii) the clearance of senescent cells (Sagiv et al., 2012), (iii) the resolution of inflammation (Thoren et al., 2012, Waggoner et al., 2012) and (iv) modulating adaptive immunity (Martin-Fontecha et al., 2004, Vitale et al., 2005). Thus, NK cell immunesenescence may have more far reaching effects upon the health of older adults than simply increasing their susceptibility to viral infection.

Here, after discussing NK cell function and the changes in NK cell biology that occur with age, we review data which suggest that in addition to the previously described association between reduced NKCC in older adults and an increased incidence of viral infection (Levy et al., 1991, Ogata et al., 1997, Ogata et al., 2001) that other features of the ageing process may be attributable in part to age-related alterations in NK function and phenotype. These include: (i) the increased reactivation rates of latent *Mycobacterium tuberculosis* (TB), (ii) reduced vaccination efficacy, (iii) slower resolution of inflammatory responses and (iv) the accumulation of senescent cells.

### 1.1 NK cell function

NK cell cytotoxicity (NKCC) and the secretion of cytokines and chemokines are the two main mechanisms NK cells use to eliminate transformed and virus-infected cells. Induction of these defensive strategies is governed by signals transmitted through germline-encoded activatory and inhibitory receptors (Lanier, 1998). Inhibitory receptors, which include members of the killer-cell immunoglobulin-like receptor (KIR) superfamily and the C-type lectin family member CD94/NKG2A, recognise self major histocompatibility complex (MHC) class I molecules and transmit inhibitory signals through an immunoreceptor tyrosine-based inhibitory motif within their cytoplasmic domain (Lanier, 1998, Pegram et al., 2011). Examples of activatory receptors are the natural cytotoxicity receptors (NCR) NKp30, NKp44 and NKp46, which recognise viral haemagglutinin (Arnon et al., 2001, Mandelboim et al., 2001) and bacterial surface proteins (Esin et al., 2008), the Fc receptor CD16, which allows NK cells to perform antibody dependent cell cytotoxicity (ADCC) and the C-type lectin family member NKG2D, whose ligands include the stress-inducible glycoproteins MHC class I-chain-related protein A (MICA) and MICB (Bauer et al., 1999).

#### 1.1.1 NKCC

**Fig. 1:**
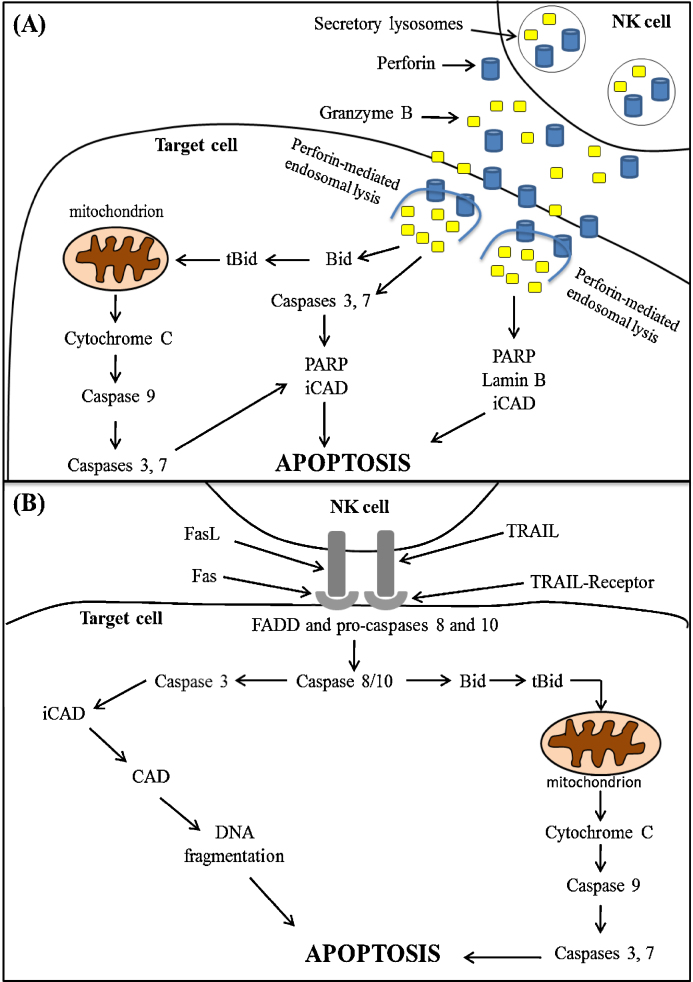
Mechanisms of natural killer cell cytotoxicity (NKCC). NK cells directly eliminate transformed cells through one of two contact-dependent mechanisms. (A) *The granule exocytosis pathway*. Following target cell recognition and activation, NK cells secrete an array of lytic effector molecules into the immunological synapse. These molecules include the pore-forming protein perforin and a family of serine proteases termed granzymes. Binding of these proteins to the target cell surface triggers their uptake into endosomes, which are subsequently lysed by perforin, resulting in the release of apoptosis-inducing granzymes into the cytoplasm (Thiery et al., 2010, Thiery et al., 2011). Shown in the figure are the 3 pathways by which granzyme B mediates target cell death. (1) As an aspase, granzyme B cleaves and directly activates effector caspases (e.g. caspases 3 and 7), which subsequently induce target cell apoptosis by degrading proteins involved in DNA repair (e.g. poly ADP ribose polymerase (PARP)) and activating the endonuclease caspase-activated DNase (CAD) by degrading its inhibitory binding partner, inhibitor of caspase-activated DNase (iCAD). (2) As well as activating caspases 3 and 7 directly, granzyme B can indirectly activate these proteases by cleaving the BH-3 family protein BH3-interacting domain (BID) death agonist into its truncated form (tBID). tBID translocates to the mitochondria, where it induces permeabilisation, leading to the release of cytochrome c. The presence of cytochrome c along with other pro-apoptotic proteins in the target cell cytosol results in the activation of the initiator caspase, caspase 9, which mediates cell death by cleaving and activating caspases 3 and 7. (3) In addition to inducing caspase-dependent target cell death, granzyme B can trigger caspase-independent cell death by directly cleaving proteins involved in DNA repair and maintenance. These proteins include PARP, inhibitor of caspase-activated DNase (iCAD) and the nuclear protein lamin B. (B) *Death receptor ligation*. Activated NK cells express on their surface Fas ligand (FASL) and TNF-related apoptotic inducing ligand (TRAIL), which bind their cognate receptors, Fas and TRAIL-R respectively on the target cell surface. Ligand binding leads to receptor oligomerisation and the recruitment of the cytosolic adaptor protein Fas-associated protein with death domain (FADD) along with the initiator caspases, pro-caspase 8 and 10 to the target cell membrane. Formation of this complex, referred to as the death-induced signalling complex (DISC), triggers the activation of caspases 8 and 10, which subsequently induce apoptosis by activating caspase 3 either directly via cleavage or indirectly by generating tBID, which leads to cell death by driving mitochondrial permeabilisation, cytochrome c release and caspase 3 activation.

NK cells directly eliminate transformed cells through two contact-dependent mechanisms: granule exocytosis and death receptor ligation (Fig. 1; Smyth et al., 2005). Of these, granule exocytosis, which is performed predominantly by CD56^DIM^ NK cells, is the main pathway by which NK cells confer host protection (Sayers et al., 1998, Smyth et al., 1999), and is characterised by the secretion of cytotoxic proteins into the immunological synapse that forms between an NK cell and its target (Fig. 1A; Smyth et al., 2005). Of the proteins released, it is the membrane-disrupting protein perforin and a family of serine proteases termed granzymes that are the critical effector molecules.

Current work suggests that after binding to phospholipid components of the target cell membrane, perforin undergoes polymerisation, triggering a membrane-repair response within the target cell that results in the co-endocytosis of membrane-bound perforin and granzymes (Thiery et al., 2010, Thiery et al., 2011). Once inside the target cell, perforin has been shown to induce endosomal lysis, leading to the release of granzymes into the cytosol (Thiery et al., 2010, Thiery et al., 2011). Human NK cells express five granzymes, namely A, B, H, K and M (Smyth et al., 2005). Of these, granzyme B has been the subject of considerable interest. As an aspartase, granzyme B cleaves proteins after aspartic acid residues. Consequently, several members of the caspase family are directly activated by granzyme B, including caspase 3 (Goping et al., 2003). This effector caspase induces apoptosis by several mechanisms, which include: (1) activating the endonuclease caspase-activated DNase (CAD) by degrading its inhibitory binding partner, inhibitor of caspase-activated DNase (iCAD) and (2) degrading proteins involved in DNA repair (e.g. poly ADPribose polymerase (PARP)) (Fig. 1A; Heusel et al., 1994, Darmon et al., 1995, Chinnaiyan et al., 1996, Taylor et al., 2008). As well as direct activation, granzyme B activates caspases 3 and 7 indirectly by driving mitochondrial permeabilisation (Fig. 1A). This occurs via granzyme B-mediated cleavage of the BH-3 family protein BH3-interacting domain (BID) death agonist into its truncated form (tBID). Once formed, tBID translocates to the mitochondria where it induces permeabilisation, leading to the release of the pro-apoptotic protein cytochrome c into the cytosol (Fig. 1A; Alimonti et al., 2001). Here, cytochrome c associates with ATP, apoptosis-activating factor 1 (Apaf-1) and pro-caspase 9, forming a structure referred to as the apoptosome (Bao and Kumar, 2007). Formation of this complex results in the activation of caspase 9, which subsequently mediates cell death by cleaving and activating caspases 3 and 7 (Fig. 1A; Bao and Kumar, 2007). In addition to mediating caspase-dependent apoptosis, granzyme B can trigger target cell death in a caspase-independent manner. This is achieved through the direct cleavage of proteins involved in DNA repair (e.g. PARP) and maintenance (e.g. lamin B) (Fig. 1A; Froelich et al., 1996, Thomas et al., 2000, Zhang et al., 2001).

Alongside granule exocytosis, NK cells directly eliminate transformed cells through death receptor engagement (Fig. 1B). In response to cytokine stimulation (Medvedev et al., 1997, Sato et al., 2001) or following ligation of activatory receptors (Wallin et al., 2003, Chua et al., 2004), NK cells express on their surface Fas ligand (FasL) and TNF-related apoptosis-inducing ligand (TRAIL), which bind to Fas and TRAIL receptor respectively on the target cell surface. This interaction results in the formation within the target cell of a death induced signalling complex consisting of the adaptor molecule Fas-associated protein with death domain (FADD) and pro-caspases 8 and 10 (Bao and Kumar, 2007). Formation of this complex leads to the activation of both caspases, which promote target cell death by activating caspase 3 either directly via cleavage or indirectly by cleaving BID into tBID, which induces mitochondrial permeabilisation and the release of cytochrome c (Fig. 1B; Lavrik et al., 2005).

#### 1.1.2 Cytokine and chemokine production

NK cells activated by cytokine stimulation (Krishnaraj and Bhooma, 1996, Mariani et al., 2001, Mariani et al., 2002b) or target cell challenge (Fauriat et al., 2010, De et al., 2011) secrete a multitude of immunoregulatory cytokines and chemokines such as tumour necrosis factor alpha (TNF-α), interferon gamma (IFN-γ), interleukin (IL)-8 and macrophage inflammatory protein-1-alpha (MIP-1α). Via the production of these soluble mediators, NK cells can amplify on-going innate immune responses by enhancing the activity of bystander cells and can also influence the early phases of an adaptive immune response by promoting dendritic cell (DC) maturation and T cell differentiation. For instance, through the secretion of IFN-γ and/or TNF-α, NK cells have been shown to not only enhance the antigen presenting capacity and cytotoxic activity of macrophages but also to drive the maturation of immature DC's (Bancroft et al., 1989, Trinchieri, 1995, Vitale et al., 2005). Furthermore, in an elegant in vivo study, Martin-Fontecha and colleagues demonstrated that after migrating to draining lymph nodes (DLN), NK cells helped drive T helper-1 (Th-1) cell polarisation by secreting IFN-γ (Martin-Fontecha et al., 2004).

### 1.2 Age-associated changes in NK cell biology

Numerous studies have investigated the effect of physiological ageing on the biology of human NK cells. In this section, we review the findings of those that have examined its impact on the composition and phenotype of the circulating NK cell pool and NK cell function.

#### 1.2.1 Effect of age on NK cell frequency and the composition of the circulating NK pool

A significant increase in the percentage and/or absolute number of CD3^−^ CD56^+^ NK cells is the general finding reported by studies that have investigated the effect of age on NK cell frequency (Lutz et al., 2005, Di et al., 1999, Le Garff-Tavernier et al., 2010, Lutz et al., 2011, Hazeldine et al., 2012). As physiological ageing is accompanied by a reduction in NK cell production and proliferation (Zhang et al., 2007), this age-related increase in NK cell number may be the result of an accumulation of long-lived NK cells in older adults (Zhang et al., 2007).

Immunogerontological studies that have examined the effect of age on the composition of the circulating NK cell pool have found a greater proportion of CD57^+^ NK cells in older adults (Lutz et al., 2005, Simpson et al., 2008, Le Garff-Tavernier et al., 2010, Hazeldine et al., 2012). As a marker of NK maturity, an increased percentage of CD57^+^ cells suggests a shift towards a more mature circulating NK pool occurs with age. With regards to NK cell subsets, studies have shown that whilst the proportions and/or number of CD56^DIM^ NK cells increase with age (Almeida-Oliveira et al., 2011, Lutz et al., 2011, Hazeldine et al., 2012) older adults possess significantly fewer CD56^BRIGHT^ NK cells (Le Garff-Tavernier et al., 2010, Almeida-Oliveira et al., 2011, Lutz et al., 2011, Hazeldine et al., 2012), resulting in a marked age-related increase in the CD56^DIM^:CD56^BRIGHT^ ratio (Krishnaraj, 1997, Hayhoe et al., 2010, Hazeldine et al., 2012). In the only study to have investigated the effect of age on the frequency of CD56^−^ NK cells, a subset that exhibits defective natural and ADCC but comparable chemokine secretion when compared to CD56^DIM^ NK cells (Hu et al., 1995, Alter et al., 2005, Mavilio et al., 2005), no age-related difference was reported (Lutz et al., 2011).

#### 1.2.2 Effect of age on NK cell phenotype

The effect of age on the expression of certain NK cell activatory receptors is controversial. Whereas some groups have reported an age-related decline in the percentage of NK cells expressing NKp30 or NKp46 (Almeida-Oliveira et al., 2011, Hazeldine et al., 2012, Tarazona et al., 2012), which in the case of NKp30 is accompanied by a reduction in its surface density (Tarazona et al., 2012), others have demonstrated no effect for age on the proportions of NK cells bearing these receptors (Le Garff-Tavernier et al., 2010). More consistent findings have however been reported for NKG2D and CD16 whose levels are maintained with age (Lutz et al., 2005, Hayhoe et al., 2010, Le Garff-Tavernier et al., 2010, Hazeldine et al., 2012). With regards to inhibitory receptors, KIR expression has been reported to be increased or unaltered with age (Lutz et al., 2005, Lutz et al., 2011, Almeida-Oliveira et al., 2011). In contrast, marked age-associated reductions in KLRG-1 and NKG2A have been documented (Lutz et al., 2005, Lutz et al., 2011, Hayhoe et al., 2010), as has a decline in CD94, the binding partner of NKG2A (Lutz et al., 2005, Hayhoe et al., 2010, Almeida-Oliveira et al., 2011, Hazeldine et al., 2012).

Recently, Bigley et al. (2012) demonstrated that infection with the latent herpes virus cytomegalovirus (CMV) has a marked impact on the surface phenotype of NK cells. The group, who used blood samples obtained solely from young donors, found that when compared to their seronegative counterparts, CMV^+^ individuals had a lower frequency of KLRG1^+^/CD57^−^ NK cells and a higher proportion of KLRG1^−^/CD57^+^ NK cells (Bigley et al., 2012), which is reminiscent of the circulating NK pool of older adults (Lutz et al., 2005, Le Garff-Tavernier et al., 2010, Hayhoe et al., 2010, Hazeldine et al., 2012). Consequently, Bigley et al. (2012) have proposed that the changes in NK cell phenotype that accompany human ageing may not be due to the ageing process per se but a greater prevalence of CMV among older individuals.

Thus, a decline in the expression of KLRG1, NKG2A and its binding partner CD94 with age has been reported by several groups. Assessing NK cell phenotype in older individuals who are CMV seronegative versus seropositive would help to provide a possible explanation for some of these changes notably in KLRG1.

#### 1.2.3 Effect of age on NKCC and ADCC

All studies published to date that have examined the effect of age on NKCC have focused exclusively upon target cell death induced by the granule exocytosis pathway, with nothing known regarding the impact of age on death receptor mediated killing. However, in spite of a focus on the granule exocytosis pathway, considerable differences in study outcome have been described, with some groups reporting a significant age-related decline in NKCC (Facchini et al., 1987, Miyaji et al., 1997, Di et al., 1999, Hazeldine et al., 2012) and others demonstrating the lytic activity of NK cells to be increased (Onsrud, 1981, Krishnaraj and Blandford, 1987, Kutza and Murasko, 1994) or unchanged (Facchini et al., 1987, Nagel et al., 1981, Almeida-Oliveira et al., 2011) with age. Inter-study differences in subject inclusion criteria and protocol design may account for these discordant findings. For example, whereas some groups have performed NKCC assays with PBMCs (Facchini et al., 1987, Nagel et al., 1981, Kutza and Murasko, 1994, Almeida-Oliveira et al., 2011), others have used peripheral blood lymphocytes (Onsrud, 1981, Di et al., 1999) or purified NK cells (Facchini et al., 1987, Miyaji et al., 1997) as their effector population. Furthermore, the studies differ in the length of time they co-cultured NK cells with their targets before assessing NKCC, the significance of which was illustrated in the work of Rukavina et al. (1998), who found NKCC exhibited an age-related decline in short-term (2 h) assays, but that after prolonged co-culture (18 h) the lytic activity of NK cells from aged donors was comparable to that of their younger counterparts.

One aspect of protocol design that was consistent throughout all of the aforementioned studies was the use of the MHC class I deficient K562 cell line as the target population. Although the cell line of choice for in vitro work, it is unlikely that the surface of every transformed cell encountered by NK cells in vivo is devoid of ligands that deliver inhibitory signals. For instance, an immune evasion strategy deployed by human CMV is to up-regulate the expression of the non-classical HLA molecule HLA-E, a ligand for the inhibitory receptor CD94/NKG2A, on the surface of infected host cells (Tomasec et al., 2000). Interestingly, it has been shown that signals transmitted to NK cells through the activatory receptor NKG2D can override those delivered by inhibitory receptors, thereby allowing NK cells to eliminate MHC class I positive targets (Bauer et al., 1999, Cerwenka et al., 2001). Whether physiological ageing has a detrimental effect upon the ability of NK cells to eliminate target cells that simultaneously deliver inhibitory and activatory signals is unknown. Performing experiments that compare the lytic activity of NK cells from young and old subjects towards target cells with varying surface expression of inhibitory receptor ligands would help address this issue.

Despite the conflicting observations described above, the general consensus within the NK cell field is that at the single cell level, NKCC is reduced with age (Facchini et al., 1987, Mariani et al., 1990, Almeida-Oliveira et al., 2011, Hazeldine et al., 2012). Indeed, an age-related increase in the proportions and/or absolute numbers of circulating NK cells was reported by many of the studies that found NKCC to be comparable between young and old donors when PBMCs were used as effector cells (Facchini et al., 1987, Mariani et al., 1990, Almeida-Oliveira et al., 2011), whereas an age-associated decrease in lytic activity has been reported by the majority of groups that have used purified NK cells at fixed concentrations in their cytolytic assays (Facchini et al., 1987, Mariani et al., 1990, Miyaji et al., 1997). Thus, the aforementioned increase in the circulating frequency of CD56^DIM^ NK cells that occurs with age (Almeida-Oliveira et al., 2011, Lutz et al., 2011, Hazeldine et al., 2012)) may represent a compensatory mechanism that counteracts the age-associated decrease in NKCC at the single cell level (Mocchegiani and Malavolta, 2004).

NK cells from older adults recognise and bind to tumour targets as effectively as those from younger subjects, suggesting the age-related impairment in NKCC is the result of a post-binding defect (Facchini et al., 1987, Ligthart et al., 1989, Vitale et al., 1992, Mariani et al., 1998). Reduced expression of perforin and the aforementioned age-associated alterations in the expression of NK cell activatory (e.g. NKp30 and NKp46) and inhibitory (e.g. KIR) receptors are two mechanisms that have been proposed to underlie the age-related decline in NKCC (Rukavina et al., 1998, Almeida-Oliveira et al., 2011). However, not all groups agree with these proposals, with some studies showing no effect for age on perforin expression (Mariani et al., 1996, Hazeldine et al., 2012) and others reporting no difference in NKCC despite marked alterations in receptor expression with age (Almeida-Oliveira et al., 2011). Recently, we showed for the first time that following target cell recognition, NK cells from older adults release less perforin into the immunological synapse when compared to NK cells from their younger counterparts, a defect we attributed to an age-related impairment in the polarisation of lytic granules to the NK target cell interface (Hazeldine et al., 2012). Underlying this impairment appears to be aberrant intracellular signalling proximal to the NK cell membrane as when NK cells are treated with phorbol 12-myristate 13-acetate (PMA) and ionomycin, two agents that bypass cell surface receptors to induce NK cell degranulation, no age-associated difference in perforin release is found (J. Hazeldine, unpublished observations). Supporting this idea, Mariani et al. (1998) have previously shown that following target cell stimulation, NK cells from older adults generate significantly less of the second messenger inositol 1,4,5-triphosphate, the result of an age-related delay in phosphatidylinositol 4,5-bisphosphate hydrolysis. Thus, we propose that reduced perforin release, due to compromised cell signalling involved in perforin granule localisation to the immune synapse, underlies the age-related decline in NKCC.

ADCC appears to be unaffected by the ageing process. Studies have shown no difference in CD16 expression with age (Hayhoe et al., 2010, Hazeldine et al., 2012) or in the ability of NK cells from old donors to lyse antibody-coated target cells (Edwards and Avis, 1979, Fernandes and Gupta, 1981, Lutz et al., 2005). However, as the NK cell pool of older adults is dominated by CD57^+^ NK cells (Lutz et al., 2005, Simpson et al., 2008, Le Garff-Tavernier et al., 2010, Hazeldine et al., 2012), which exhibit potent ADCC (Lopez-Verges et al., 2010), it may be that similar to natural cytotoxicity, ADCC is also impaired at the single cell level with age.

#### 1.2.4 Effect of age on NK cell migration

A crucial step in NK cell-mediated defence is their migration through tissue to the site of inflammation, where upon arrival they recognise, bind and eliminate their targets. Results of two recent murine studies suggest that NK cell migration is impaired with age. In a model of influenza infection, Beli et al. (2001) found significantly lower numbers of NK cells in the lungs and spleen of aged mice at both day two and day four post infection when compared to the corresponding samples from young mice. This observation was in agreement with earlier work that had reported an age-associated decline in the recruitment of NK cells to draining lymph nodes following viral challenge (Fang et al., 2010). In this latter study, defective NK cell migration was partly attributed to significantly reduced expression of L-selectin, a molecule critical for lymphocyte homing to lymph nodes (Fang et al., 2010).

To our knowledge, no study has been performed that has directly assessed the impact of human ageing on NK cell migration. However, some groups have examined its effect on the expression of surface receptors known to be involved in this process. No age-related differences have been observed in the expression of the adhesion receptor CD2 (Hayhoe et al., 2010) or the chemokine receptors CCR3 or CCR5 (Mariani et al., 2002a, Mariani et al., 2002b). In contrast, the density of CXCR1, a receptor for the chemokine IL-8, has been shown to be markedly lower on the surface of NK cells from older adults (Mariani et al., 2002a), although no effect for age was found when the frequency of CXCR1^+^ NK cells was measured (Mariani et al., 2001, Mariani et al., 2002a).

Further research is clearly required in order to gain a greater understanding of the impact ageing has on NK cell migration. However, based on existing data, it appears that this function of NK cells is reduced with age, which when combined with the aforementioned impairment in NKCC, would be expected to compromise their ability to eliminate cellular targets in vivo.

#### 1.2.5 Effect of age on the response to and production of cytokines and chemokines

Cytokine stimulation markedly enhances NKCC. In vitro this is assessed by the ability of cytokine-activated NK cells to induce lysis of the NK cell resistant Daudi cell line. Cytokine-activated NK cells from older adults exhibit greater cytotoxicity against Daudi target cells than their untreated counterparts indicating that NK cells from older adults are responsive to cytokine treatment (Kutza and Murasko, 1996, Krishnaraj, 1997, Mariani et al., 2001). However, whether the level of cytotoxicity achieved is different to that of cytokine-activated NK cells from younger adults is currently unclear (Kutza and Murasko, 1994, Kutza and Murasko, 1996, Krishnaraj and Bhooma, 1996, Krishnaraj, 1997, Mariani et al., 2001).

NK cells isolated from old subjects respond to cytokine stimulation by up-regulating their production of IFN-γ, MIP-1α and IL-8 (Krishnaraj and Bhooma, 1996, Krishnaraj, 1997, Mariani et al., 2001, Mariani et al., 2002b). However, the levels generated are significantly lower than those produced by NK cells from younger subjects (Krishnaraj and Bhooma, 1996, Mariani et al., 2001, Mariani et al., 2002a, Mariani et al., 2002b). As it is the CD56^BRIGHT^ NK cell subset that primarily responds to cytokine challenge (Cooper et al., 2001b, Fehniger et al., 2003, Ferlazzo et al., 2004, Anfossi et al., 2006), one explanation for this impaired response could be the aforementioned age-related decline in CD56^BRIGHT^ NK cells (Le Garff-Tavernier et al., 2010, Almeida-Oliveira et al., 2011, Lutz et al., 2011, Hazeldine et al., 2012). Recently, target cells have been shown to trigger cytokine and chemokine production by NK cells (Fauriat et al., 2010, De et al., 2011). In a small pilot study, we have found the amount of IFN-γ, MIP-1α and IL-8 but not TNF-α produced by target cell-stimulated NK cells from older adults is significantly lower than the levels secreted by NK cells from younger subjects (J. Hazeldine, unpublished observations). These results along with the aforementioned cytokine data suggest the immunoregulatory capacity of NK cells is reduced with age.

### 1.3 NK cell immunesenescence and its impact upon healthy ageing

**Fig. 2:**
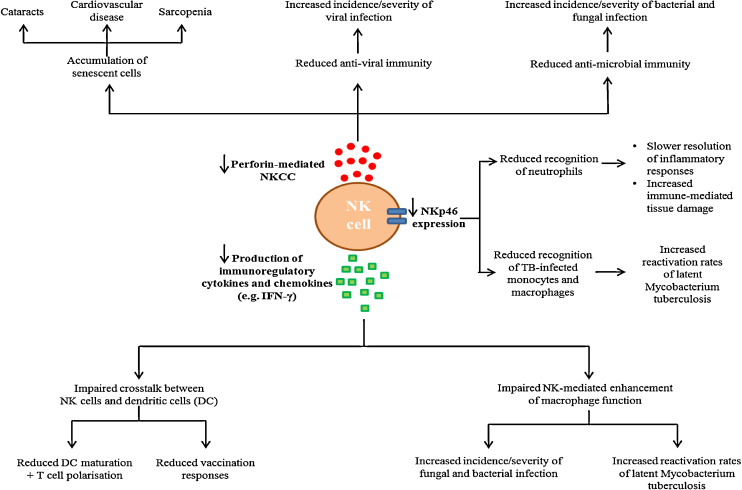
Features of the ageing process proposed to result in part from age-associated changes in NK cell biology. It is hypothesised that alongside the previously described association between decreased NKCC and an increased susceptibility to viral infection in older subjects (Levy et al., 1991, Ogata et al., 1997, Ogata et al., 2001) that the age-related reduction in perforin-mediated cytotoxicity and cytokine/chemokine production along with changes in NK cell surface phenotype have additional consequences for the health of older adults. These are proposed to include: (1) the accumulation of senescent cells and the subsequent development of such age-related pathologies as sarcopenia and cardiovascular disease (Sagiv et al., 2012), (2) slower resolution of inflammatory responses and increased immune-mediated tissue damage due to impaired NK-mediated elimination of neutrophils, (3) increased reactivation rates of latent *Mycobacterium tuberculosis* (TB) due to impaired production of IFN-γ by NK cells and reduced recognition of TB-infected monocytes and macrophages by the activating receptor NKp46 and (4) poorer vaccination responses as a result of impaired NK cell-dendritic cell (DC) cross-talk due to reduced IFN-γ production by activated NK cells.

Given the significant role of NK cells in anti-viral defence (Biron et al., 1989, Etzioni et al., 2005, Eidenschenk et al., 2006), studies that have examined the impact of NK cell immunesencene on the health of older adults have to date focused primarily on their response to viral challenge, reporting that decreased NKCC in these individuals is associated with a past history of severe infection, an increased incidence of infectious disease and a reduced probability of survival following infection (Levy et al., 1991, Ogata et al., 1997, Ogata et al., 2001). However, in light of recent work that suggests the role of NK cells extends beyond anti-viral immunity into such areas as the resolution of inflammation (Thoren et al., 2012, Waggoner et al., 2012) and the recognition and elimination of senescent cells (Sagiv et al., 2012), it is likely that NK cell immunesenescence has wider implications on the health of older adults than first thought. Indeed, it has been proposed that in addition to increasing the susceptibility of older adults to viral infection (Levy et al., 1991, Ogata et al., 1997, Ogata et al., 2001), age-associated changes in NK cell function may be responsible in part for the increased frequency of senescent cells found in aged tissue (Sagiv et al., 2012) and the poorer adaptive immune responses elicited by aged subjects (Tarazona et al., 2012). In this section, as well as discussing these ideas, we propose NK cell immunesenescence may underlie other features of the ageing process such as the increased reactivation rates of *M. tuberculosis*, the slower resolution of inflammatory responses and the increased incidence of bacterial and fungal infection (Fig. 2).

#### 1.3.1 Accumulation of senescent cells

A feature of physiological ageing is the appearance of senescent cells. These cells, which have been detected in skin (Dimri et al., 1995), bone (Price et al., 2002) and endothelium (Minamino et al., 2002) from older adults, reside in a state of irreversible cell cycle arrest, yet remain metabolically active, secreting an array of growth factors, pro-inflammatory cytokines and proteases. Recently, evidence has emerged that suggests that by compromising tissue homeostasis and function, senescent cell accumulation contributes to the development of several age-associated pathologies such as sarcopenia and cataracts (Baker et al., 2011).

The immune system is involved in the recognition and elimination of senescent cells. In different experimental settings, macrophages, neutrophils, NK cells and T cells have all been implicated in the clearance of senescent cells (Xue et al., 2007, Krizhanovsky et al., 2008, Kang et al., 2011). In a recent article, Sagiv and co-workers demonstrated that NK-mediated elimination of senescent cells occurs exclusively through the granule exocytosis pathway (Sagiv et al., 2012), a finding that led the group to speculate that an age-related decline in perforin-mediated NKCC may be responsible in part for the increased frequency of senescent cells found in aged tissue (Dimri et al., 1995, Minamino et al., 2002, Price et al., 2002, Sagiv et al., 2012). Recently, we have shown that when compared to those isolated from younger subjects, NK cells from older adults release significantly less perforin into the immunological synapse that is formed following target cell contact (Hazeldine et al., 2012), a defect, which based on the findings of Sagiv et al. (2012) would be expected to hamper the ability of NK cells from older adults to remove senescent cells.

#### 1.3.2 Impaired crosstalk between the innate and adaptive arms of the immune system

It is now recognised that the innate and adaptive arms of the immune system undergo considerable cross-talk and reciprocal interaction. Via direct cytotoxicity and cytokine production, NK cells find themselves at the interface of this relationship by influencing DC maturation and T cell differentiation.

NK cell-DC cross-talk has two outcomes for DCs. Following direct contact and the recognition of an unknown ligand on the DC surface by the NCR NKp30, activated NK cells have been shown to: (i) directly lyse iDCs, which may be a means by which NK cells limit iDC activation at sites of antigenic challenge (Ferlazzo et al., 2002, Moretta, 2002), and (ii) secrete IFN-γ and TNF-α, thereby promoting DC maturation (Vitale et al., 2005). As well as influencing a T cell response indirectly by modulating the maturity status of DCs, NK cells, in times of infection have been shown to migrate to T cell zones of DLN, where via IFN-γ secretion, they drive Th-1 cell polarisation (Martin-Fontecha et al., 2004).

With age, expression of NKp30 on the NK cell surface is reduced (Tarazona et al., 2012; J. Hazeldine, unpublished observations), as is the level of granule-mediated cytotoxicity and the amount of IFN-γ produced by stimulated NK cells from older adults (Facchini et al., 1987, Krishnaraj and Bhooma, 1996, Miyaji et al., 1997, Di et al., 1999, Hazeldine et al., 2012; J. Hazeldine, unpublished observations). Thus, as well as adversely affecting the immediate response to transformed cells, NK cell immunesenescence may have a negative impact upon the development of an adaptive immune response. On this note, Tarazona et al. (2012) have proposed that the reduced expression of NKp30 they observed on the surface of aged NK cells would have a detrimental effect upon NK-DC crosstalk, leading to a less effective adaptive immune response against virus-infected and malignant cells (Tarazona et al., 2012). Moreover, it is reasonable to think that as a result of reduced IFN-γ secretion, NK cells from older adults would be less efficient at driving DC maturation and T cell polarisation. Interestingly, work by Mysliwska et al. (2004) has shown that the generation of protective anti-hemagglutinin titres and the production of Th-1 cell cytokines following influenza vaccination is markedly lower in older adults with low NK activity when compared to those with high NK activity, which was associated with a greater incidence of respiratory tract infection in the former group (Mysliwska et al., 2004). This inadequate response to vaccination may be related in some part to the aforementioned changes in NK biology adversely affecting the ability of these cells to influence the early phases of adaptive immunity.

#### 1.3.3 Delayed resolution of inflammatory responses

An unexpected role for NK cells in immune regulation has recently emerged. In murine models of viral infection, NK cells have been shown to lyse DCs as well as CD4^+^ and CD8^+^ T cells in a perforin-dependent manner (Andrews et al., 2010, Waggoner et al., 2012, Lang et al., 2012), whilst in vitro, NK cells induce neutrophil apoptosis by death receptor ligation, which requires recognition of an as of yet unidentified ligand on the neutrophil surface by the NCR NKp46 (Thoren et al., 2012).

In aged individuals, the frequency of NKp46^+^ NK cells is reduced (Almeida-Oliveira et al., 2011, Hazeldine et al., 2012), which we expect would lead to reduced neutrophil recognition by NK cells. Whether NK cell lysis induced by death receptor ligation is altered with age is currently unknown. However, reduced interaction between NK cells and neutrophils as a result of a decline in NKp46 expression would in our opinion result in reduced rates of NK-mediated neutrophil apoptosis, consequences of which could be the slower resolution of inflammatory responses that has been reported in older adults (Boyd and Orihuela, 2011), and the onset of neutrophil necrosis, which would lead to local tissue damage. On this note, the aforementioned lysis of CD4^+^ T cells by NK cells has been proposed in a model of viral infection to promote host survival by preventing immune-mediated pathology (Waggoner et al., 2012). Thus, the decrease in perforin-mediated NKCC that we have shown occurs with age (Hazeldine et al., 2012) suggests that the immune regulatory function of NK cells may be impaired. This may contribute to the increased severity of infection in older adults, along with the longer resolution phase and recovery time following infection.

#### 1.3.4 Increased reactivation rates of *M. tuberculosis* (TB)

Based on in vitro studies that have shown NK cells lyse TB-infected monocytes and macrophages, and generate significant amounts of IFN-γ when exposed to these infected phagocytes (Vankayalapati et al., 2002, Vankayalapati et al., 2005), NK cells are thought to be involved in the immune response against this intracellular bacterium. With age, reactivation rates of latent TB increase (Horsburgh et al., 2010), suggesting that the immune response towards this pathogen may be altered in older adults. Since an age-related decrease in the frequency of NK cells that express NKp46, an activating receptor involved in the recognition of TB-infected monocytes and macrophages (Vankayalapati et al., 2002, Vankayalapati et al., 2005), has been reported (Almeida-Oliveira et al., 2011, Hazeldine et al., 2012), along with a reduction in IFN-γ secretion by NK cells from older adults (Krishnaraj and Bhooma, 1996; J. Hazeldine, unpublished observations), then a weakened NK response may contribute to this greater rate of TB reactivation in older adults (Horsburgh et al., 2010).

#### 1.3.5 Reduced anti-microbial immunity

Although renowned for their anti-viral properties, the role of NK cells in host defence is not limited to just protecting against infection with these intracellular pathogens. Through perforin-mediated cytotoxicity and the secretion of IFN-γ, NK cells have been implicated in the immune response against bacteria and fungi, either by directly targeting these pathogens for lysis or by enhancing the activity of bystander immune cells (Scott et al., 2003, Ma et al., 2004, Sporri et al., 2006, Schmidt et al., 2011).

Compared to younger subjects, older adults report an increased incidence of bacterial and fungal infection (Marston et al., 1997, Kauffman, 2001, Janssens and Krause, 2004). Although an age-associated decline in the function of neutrophils, which represent the first-line of defence against these pathogens, has been proposed as the major factor underlying this increased rate of infection (Lord et al., 2001, Wessels et al., 2010), NK cell immunesenescence may also be a contributory factor. The decrease in NKCC mediated by the granule exocytosis pathway that occurs with age would reduce the anti-fungal activity of NK cells (Facchini et al., 1987, Miyaji et al., 1997, Di et al., 1999, Hazeldine et al., 2012), whereas the age-related decline in IFN-γ secretion (Krishnaraj and Bhooma, 1996; J. Hazeldine, unpublished observations) would have a negative impact on bacterial clearance, as through the secretion of this cytokine, NK cells have been shown to enhance the anti-bacterial activity of tissue-resident macrophages (Scott et al., 2003). Thus, reduced NK cell function may compromise bacterial as well as viral immunity.

### 1.4 Conclusions

Recent studies in the field of NK cell research have shown the function of these innate lymphocytes extends beyond their well-documented role in anti-viral and tumour immunity into such areas as immune regulation, the initiation of adaptive immune responses, anti-microbial immunity and the clearance of senescent cells. Thus, besides the much publicised increase in viral infection rates, several features of the ageing process such as the reduced efficacy of vaccination, the appearance of senescent cells and the higher rates of fungal infection may be attributable in part to the decline in NK cell function that accompanies human ageing. If true, then developing strategies to prevent, delay or reverse NK cell immunesenescence may be one way by which to improve the health of older adults.
